# Visceral Leishmaniasis as a Possible Reason for Pancytopenia

**DOI:** 10.3389/fped.2015.00059

**Published:** 2015-06-29

**Authors:** Kira-Lee Koster, Hans-Jürgen Laws, Anja Troeger, Roland Meisel, Arndt Borkhardt, Prasad Thomas Oommen

**Affiliations:** ^1^Department of Pediatric Oncology, Hematology and Clinical Immunology, Medical Faculty, Center of Child and Adolescent Health, Heinrich-Heine University Düsseldorf, Düsseldorf, Germany; ^2^Department of Pediatric Hematology and Oncology, Center for Pediatrics, University of Bonn, Bonn, Germany

**Keywords:** pancytopenia, visceral leishmaniasis, recurrent fever, hepatosplenomegaly, children

## Abstract

Leishmaniasis is caused by different species of the protozoa, *Leishmania*, and frequently found in South-Western Asia, Eastern Africa, Brazil, and Mediterranean countries. *Leishmania* are transmitted to humans by the bite of sandflies. After weeks to months, unspecific symptoms may occur, accompanied by more specific findings like pancytopenia and organomegaly. We report two children with pancytopenia and hepato-/splenomegaly: a 1-year-old boy was first diagnosed with an *Adenovirus*-infection, accompanied by fever, pancytopenia, and hepatosplenomegaly who had spent his summer vacation in Spain and a 3-year-old boy of Macedonian origin who was first diagnosed with a *Parvovirus B19-*infection again accompanied by splenomegaly and pancytopenia. In both children, leukemia was excluded by an initial bone marrow puncture. As fever was still persistent weeks after the children’s first hospital stay, both children received antibiotics empirically without sustainable effect. While different autoantibodies were present in both children, an immunosuppressive therapy was initiated in the younger boy without therapeutic success. A second bone marrow puncture was performed and *Leishmania* were finally detected morphologically and proven serologically. After weight-adjusted treatment with liposomal Amphotericin B for 10 days, both children recovered completely without relapse. Aim of this report is to broaden the spectrum of differential diagnoses in children with pancytopenia, splenomegaly, and fever to visceral leishmaniasis particularly when travel history is positive for the Mediterranean area. The infection may mimic more common diseases, such as leukemia, viral infections, or autoimmune diseases, because polyclonal B cell activation and other mechanisms may lead to multiple positive serologic tests. Both cases illustrate typical pitfalls and shall encourage taking Leishmaniasis into diagnostic consideration.

## Introduction

We would like to discuss two cases where children presented with fever, pancytopenia, and hepatosplenomegaly with a focus on infectious, autoimmune, and malignant differential diagnoses.

### Case 1

**Figure 1:**
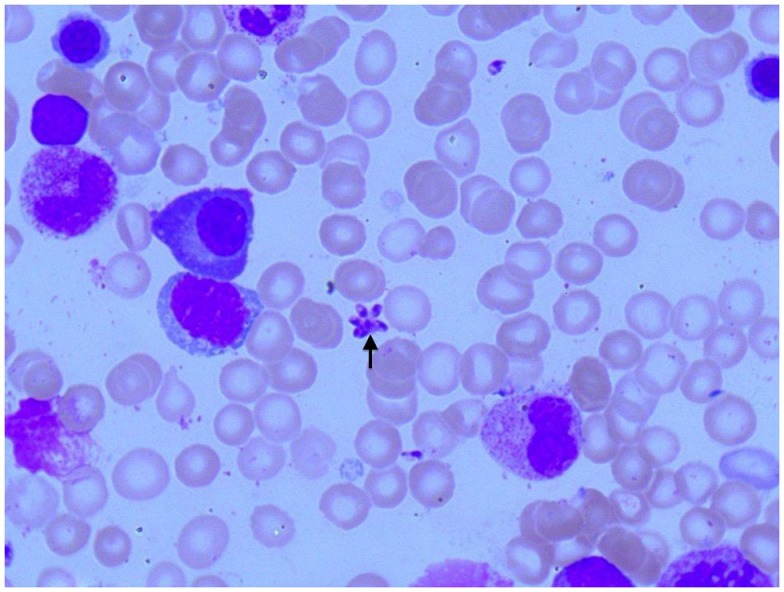
**Photomicroscopy of the first bone marrow puncture in patient A (Pappenheim’s staining, 100× magnification): *Leishmania* are difficult to detect and subtle (arrowhead indicates *Leishmania*)**.

**Figure 2:**
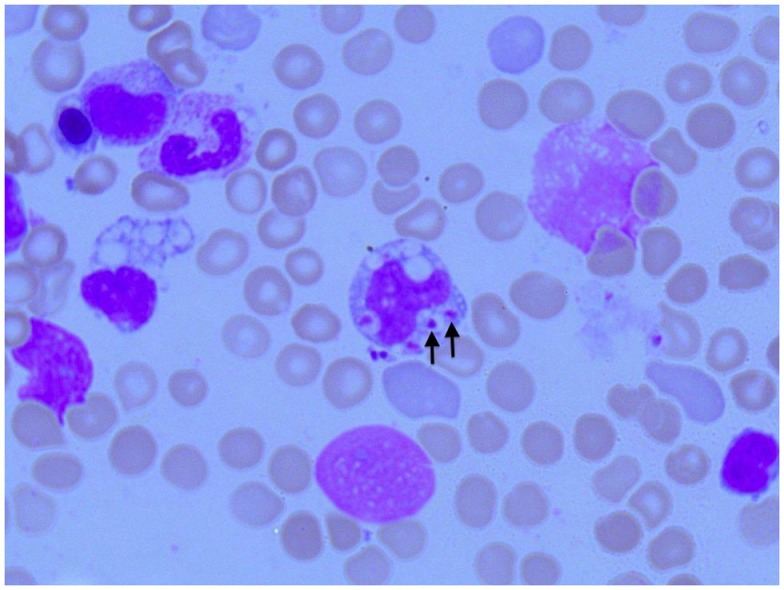
**Photomicroscopy of the first bone marrow puncture in patient A (Pappenheim’s staining, 100× magnification): *Leishmania* can also be seen intracellular in the first smear (arrowheads indicate *Leishmania*)**.

**Figure 3:**
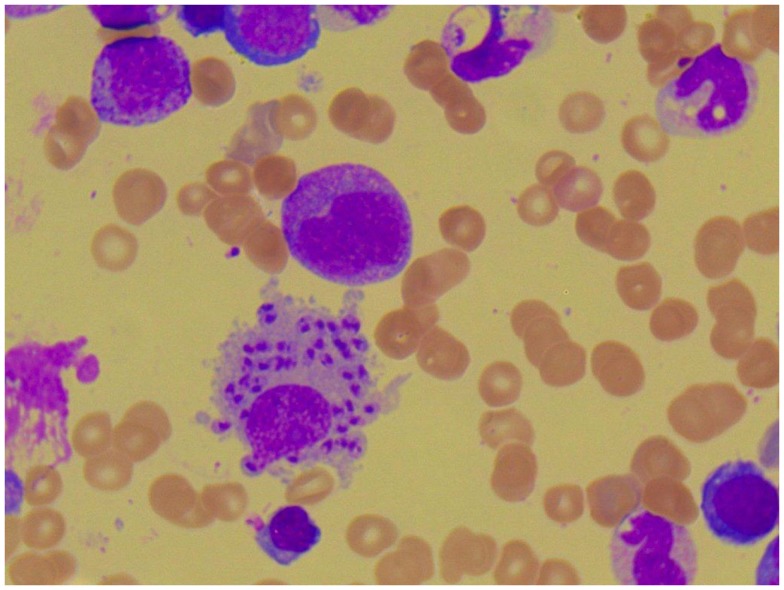
**Photomicroscopy of the second bone marrow puncture in patient A (Pappenheim’s staining, magnification 100×): numerous *Leishmania* species are clearly visible intra- and extracellularly**.

**Figure 4:**
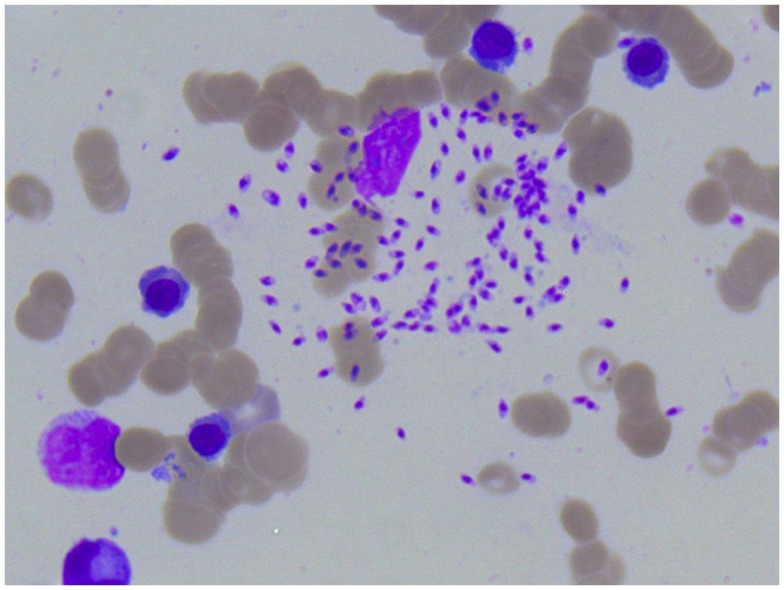
**Photomicroscopy of the second bone marrow puncture in patient A (Pappenheim’s staining, magnification 100×): numerous *Leishmania* species are clearly visible intra- and extracellularly**.

A 1-year-old boy (patient A) was referred to our hospital because of a persistent pancytopenia. He was treated initially because of an *Adenovirus* infection accompanied by recurrent fever, rhinitis, and cough. Apart from hepatosplenomegaly, the clinical examination at our hospital was unremarkable; the boy was in satisfying general condition. Laboratory findings encompassed a positive *Adenovirus* PCR in peripheral blood, bone marrow, and feces, and a conspicuous complete blood count indicating leukopenia, thrombocytopenia, anemia as well as an elevated C-reactive protein (CrP) level at first. To exclude acute leukemia, a bone marrow puncture was performed. *Leishmania* were already visible in the first blood smears but were not recognized as *Leishmania* at that point. Hence, Leishmaniasis was not taken into diagnostic consideration at first and the bone marrow therefore falsely interpreted as being normal (Figures 1 and 2). A positive direct Coombs test and antibodies against neutrophil granulocytes as well as against platelets were detected later on (Table 1). Despite the positive direct Coombs test, no further signs of hemolysis could be detected. In the absence of other explanations, pancytopenia was interpreted as most likely being virus-associated. The boy was discharged in stable condition. Yet, even weeks after the first hospital stay, fever, pancytopenia, and hepatosplenomegaly were still present. Although no infectious focus was found, an empirical antibiotic therapy was started without sustainable success. Because of the presence of antibodies against neutrophils, platelets, and a positive direct Coombs test, Evans syndrome was considered in the further course, and an immunosuppressive therapy with prednisolone and mycophenolate mofetil was initiated. Despite a slight increase in leukocytes and hemoglobin, pancytopenia, recurrent fever, and hepatosplenomegaly were still persistent 3 months after the child’s first hospital admission. Due to the unspecific laboratory findings and sustained pancytopenia, a second bone marrow puncture was performed. Here, parasites suspicious for *Leishmania* were finally detected morphologically and later on *Leishmania* species of the *L. donovani* complex (*L. donovani, L. infantum*, and *L. chagasi*) were proven serologically (Figures 3 and 4). Six months before the first symptoms had occurred, the boy had spent the summer vacation in Spain together with his parents and his twin brother. An intravenous therapy with liposomal Amphotericin B (2.8 mg/kg/day) was administered for 10 days. Following this treatment, the boy recovered completely and showed no relapse so far. The blood count fully recovered almost 2 months after the end of the therapy; hepatosplenomegaly resolved 9 months after the end of therapy.

**Table 1:** **Clinical and laboratory findings at admission/during the first stay**.

	Patient A (1-year-old boy)	Patient B (3-year-old boy)
Clinical findings at admission	Hepatosplenomegaly, recurrent fever	Hepatosplenomegaly, recurrent fever, sweats
Measurements in the abdominal ultrasound at admission/maximum sizes (measured in the anterior axillary line)	Liver 10.9 cm/11.5 cm (upper normal value: 8.9 cm) Spleen 10.2 cm/12.1 cm (upper normal value: 5.2 cm)	Liver 9.8 cm/11.5 cm (upper normal value: 10.72 cm) Spleen 11.2/14.9 cm (upper normal value: 6.74 cm)

Blood count at admission	Leukocytes 5500/μl (6000–18000)	Leukocytes 6600/μl (5000–17,000)
	Erythrocytes 3.3 Mio/μl (3.8–5.3)	Erythrocytes 3.29 Mio/μl (4.2–5.5)
	Platelets 17,000/μl (130,000–170,000)	Platelets 127,000/μl (130,000–170,000)
	Hemoglobin 7.0 g/dl (10.2–13.4)	Hemoglobin 7.7 g/dl (11.1–13.9)
	Hematocrit 23.6% (31–40)	Hematocrit 23.0% (33–41)
	Mean corpuscular volume 72.4 fl (70–86)	Mean corpuscular volume 69.9 fl (75–87)
	Mean corpuscular hemoglobin 21.5 pg (23–31)	Mean corpuscular hemoglobin 23.4 pg (20–32)
	Red cell distribution width 18.1% (11.6–14.6)	Red cell distribution width 14.8% (11.6–14.6)
	CrP 7.1 mg/dl (<0.5)	CrP 2.7 mg/dl (<0.5)
	Reticulocyte index 6.6% (0.5–2)	Reticulocyte index 5.4% (0.5–2)
	Reticulocytes 238,000/μl (22,000–112,000) (4 weeks after first admission)	Reticulocytes 176,000/μl (22,000–112,000)

Virology/microbiology at admission	Anti-*HHV6*-IgG positive	*Parvovirus B19* IgG + IgM positive
	Anti-*HHV6*-IgM unspecific (blood)	*Parvovirus B19*-PCR positive (255,623 IU/ml)
	*Adenovirus*-DNA-PCR positive [respiratory swab (12,100 Copies/ml), bone marrow (15,800 Copies/μg-DNA), and blood (10,700 Copies/μg-DNA)]	Anti-*CMV* -IgG positive
		Anti-*HHV6*-IgG positive
		Anti-*HHV6-*IgM unspecific (blood)

Autoantibodies	Positive direct Coombs test	Positive direct Coombs test
	Antibodies against neutrophils	
	Antibodies against platelets	

Immunoglobulin	IgG 1890 mg/dl (232–1411)	IgG 2670 mg/dl (500–1300)
	IgA 111 mg/dl (<83)	IgA 97 mg/dl (40–180)
	IgM 286 mg/dl (<145)	IgM 196 mg/dl (40–80)

The boy’s twin brother, who had been on the same vacation and had spent the entire time close to his brother, showed no symptoms or blood count irregularities at any time and no *Leishmania* antibodies were detected serologically.

### Case 2

A 3-year-old boy of Macedonian origin (patient B) was referred to our hospital due to a palpable resistance in the left upper abdomen and a microcytic anemia. On clinical examination, an isolated splenomegaly was found (Table 1). No further clinical findings could be detected. In the laboratory results, slightly lowered platelets and erythrocytes were present while the white blood counts remained normal at the beginning, yet deteriorated in the course of the disease resulting in pancytopenia. A positive direct Coombs test also without signs of hemolysis could be detected here. Viral serology was positive for *Parvovirus B19* (Table 1). Consecutively, a bone marrow puncture was performed without pathological findings. The *Parvovirus B19* infection was considered the most likely reason of pancytopenia. Four weeks after the first hospital admission, the boy returned with recurrent fever and sweating. Because of high fever, an empirical antibiotic therapy was performed yet without success. Pancytopenia and splenomegaly were still persistent and progressive, accompanied by hepatomegaly. Conspicuous laboratory findings were a positive direct Coombs test as well as a positive *Hanta-Virus* IgM. A second bone marrow puncture was performed and now parasites suspicious for *Leishmania* could be detected and later on species of the *L. donovani* complex were confirmed serologically. Treatment with liposomal Amphotericin B (2.7 mg/kg/day) was performed for 10 days and the boy recovered without relapse. Three months after the initial admission, *Hanta-Virus* IgM as well as *Hanta-Virus* IgG was negative.

## Background

Visceral leishmaniasis is an infection caused by different species of the protozoa *Leishmania*. *Leishmania* are transmitted to humans by the bite of sandflies. After weeks to months, unspecific symptoms, such as fever, loss of appetite, weight loss, and lymphadenopathy, may occur, often accompanied by more specific findings, such as pancytopenia and hepatosplenomegaly (1–3). In addition, different autoantibodies against cellular and humoral components may occur (4, 5). In the described cases here, pancytopenia was the most striking finding, which lead to different diagnostic procedures. In children with pancytopenia, first of all, common bone marrow disease, such as leukemia, have to be considered. More seldomly, diseases, such as myelodysplastic syndrome or aplastic anemia, are being found. Furthermore, multiple viral agents, such as EBV, CMV, HIV, and in rare cases also Rubella, Influenza, Parainfluenza, and Hepatitis A can lead to pancytopenia. Finally, toxic substances, such as cytostatic drugs, irradiation, and severe vitamin B 12 deficiency, have to be taken into consideration when pancytopenia occurs in children. A bone marrow puncture is needed to clarify further diagnostic and therapeutic steps.

Leishman and Donovan first described visceral leishmaniasis in 1903. Manifestations of leishmaniasis comprise cutaneous leishmaniasis, characterized by local cutaneous lesions, mucosal leishmaniasis, affecting the oral mucosa and the mucosa of the upper airways, and visceral leishmaniasis with a potentially fatal course if untreated. Leishmaniasis is a globally distributed disease observed in more than 80 countries worldwide. Although the majority of cases occur in countries, such as Bangladesh, Brazil, India, and Sudan (6), leishmaniasis is also endemic, e.g., in the Mediterranean Area. In this area, Morocco, Italy, Albania, Algeria, and Spain reported the highest number of cases of visceral leishmaniasis (7). Thus, visceral leishmaniasis remains no longer a merely tropical disease, but must be taken into consideration also in Germany with many travelers returning from the Mediterranean area every year (8, 9). Once diagnosed, liposomal amphotericin B is an effective drug with only mild side effects (10, 11). Oral miltefosine, an antiprotozoal alkylphosphocholine agent, might be the first choice in developing countries due to lower costs in comparison to liposomal Amphotericin B and has been proven to be effective as well (12).

## Discussion

We report and discuss two patients who presented with fever, pancytopenia, and organomegaly. After initially misleading laboratory results in respect of viral or even autoimmune etiology, visceral leishmaniasis was finally diagnosed after performing a second bone marrow examination. A successful antimicrobial therapy was finally initiated.

In our department of Pediatric Oncology, Hematology, and Clinical Immunology, a total of two patients were diagnosed with visceral leishmaniasis in the past 10 years. In comparison, 266 cases of hematologic neoplasias (acute lymphoblastic leukemia, acute myeloid leukemia, chronic myeloid leukemia, myelodysplastic syndrome, juvenile myelomonocytic leukemia) and severe aplastic anemia were diagnosed in our department between 2005 and 2014, which is an average of 26 per year. Thus, with an average of 0.2 cases per year, visceral leishmaniasis remains a very rare disease. Diagnosis of this infection can be very challenging for several reasons.

Both cases illustrate that the correct diagnosis of leishmaniasis may be delayed up to months after first symptoms occur. Unspecific autoimmune findings, such as detection of autoantibodies, may lead to misdiagnosis of an autoimmune disease. Especially in patient A, the detected antibodies against platelets and neutrophils as well as the positive direct Coombs test mislead and raised suspicion for an autoimmune disease.

Also, in the cases presented here, initial bone marrow analyses failed to detect *Leishmania* even though retrospectively isolated parasites where already visible here. Moreover, both patients obviously suffered from concurrent viral infections. In patient A, pancytopenia was expected to be caused by the *Adenovirus* infection. In patient B, a *Parvovirus* B19-infection was detected. Although mostly transient aplastic anemia occurs due to a *Parvovirus* B19-infection in the bone marrow, persistent pancytopenia and severe aplastic anemia have also been described for immunocompetent and immunocompromised patients infected with *Parvovirus B19* (13–16). Leishmaniasis may mimic autoimmune cytopenias like Evans syndrome or be associated with viral infections but it may also lead to and mimic less frequent diseases, such as macrophage activation syndrome (MAS) (17, 18) or systemic lupus erythematosus (19). MAS is characterized by T cell and macrophage activation, leading to cytokine overproduction resulting in inflammation. Due to increased macrophage activation, blood cells may be phagocytosed consecutively leading to cytopenia (17). In a very recent study, it has been shown that autophagocytosis may play an important role in the immune response to *Leishmania*. Whereas other infections may trigger macrophage activation and T cell response, an infection with vital and dead *Leishmania* at the same time may induce autophagocytosis in cells infected with dead *Leishmania* resulting in a hampered adaptive immune response, so vital *Leishmania* will not be killed at all. In cells only infected with vital *Leishmania*, the immune response is not hampered due to the missing induction of autophagocytosis. According to these study results, an infection solely with vital *Leishmania* will not lead to autophagocytosis and the immune response kills the entered *Leishmania* (20). Several investigators have revealed the presence of autoantibodies against cellular and humoral components as well as against nuclear antigens (4, 5) in visceral leishmaniasis. Many different autoantibodies/laboratory values might be altered, such as antinuclear antibodies (ANA), rheumatic factor, anti-cardiolipin antibodies, cryoglobulins, Coombs test, hypergammaglobulinemia, anti-smooth muscle antibodies (ASMA), protoplasmic-staining anti-neutrophil cytoplasmic antibodies (p-ANCA), anti-extractable nuclear antigens antibodies (anti-ENA), anti-myeloperoxidase antibodies (anti-MPO), anti-Smith antibodies (anti-SM), anti-Sjögren’s-syndrome-related antigen A (anti-SS-A)/anti-Ro antibodies (anti-Ro), anti-SS-B/anti-La, anti-ribonucleoprotein antibodies (anti-RNP), and decreased C3 and C4 (4, 5, 21). Liberopoulos et al. showed that all autoimmune laboratory findings normalized only 3 months after therapy (21). Therefore, it is important to keep in mind that leishmaniasis might resemble autoimmune diseases in a sense that autoantibodies may be detectable as presented here. The presence of these unspecific autoantibodies may be explained in part by polyclonal B-cell-activation, which may lead to hypergammaglobulinemia (4, 22, 23). Of note, both children in our cases showed elevated immunoglobulin G levels during their first hospital stay. In patient B, we detected a positive *Hanta-Virus* IgM beside the unspecific autoimmune findings, which was negative 3 months after initial admission. The *Hanta-Virus* IgG has never been positive. It cannot be excluded that these antibodies were positive due to polyclonal B-cell activation, but it seems to be more likely that the *Hanta-Virus-*IgM was positive due to cross-reacting antibodies.

Our reported cases underline the importance of thorough history taking regarding past travel history. Especially in Western countries, it is important to remember that leishmaniasis is not only a tropical disease but also endemic in some of our favorite travel destinations in the Mediterranean area (7). A recent multi-centre study reported at least 10 travelers who visited endemic areas in Europe between 2000 and 2012 and developed visceral leishmaniasis. The visited countries were – amongst others – Spain, Portugal, Greece, and Macedonia (24). Several investigators have shown that infections acquired in the Mediterranean area are mostly caused by species of the *L. donovani* complex, especially *L. infantum* (24–27). In our reported cases, species of the *L. donovani* complex were detected as well. The latest reported outbreaks of leishmaniasis in Spain (2009, caused by *L. infantum*) and Italy (2012) demonstrate that leishmaniasis is a current challenge that needs to be faced (26, 28).

Therefore, Smith et al. underline the importance of thorough history taking especially with regard to holidays spent in the Mediterranean basin as visceral leishmaniasis can no longer be considered to be an “exotic” disease (29). Leishmaniasis is characterized by a long incubation period of weeks to months, seldom years (30). Thus, due to the long interval between travel activity and onset of symptoms, important stays abroad may not be reported in first place. This may add to a delay of the correct diagnosis (29). In our reported cases, patient A had his last stay abroad in an endemic region 6 months before disease onset (vacation in Spain), patient B at least 1 year before (vacation in Macedonia). Although it is often stated that visceral leishmaniasis affects especially young children, in different studies dealing with visceral leishmaniasis acquired in Europe, patients affected seem to be adults. In a study from Italy with a 22 years span, seven patients with visceral leishmaniasis were identified, six of them were adults, and only one child was reported (25). In the EuroTravNet multi-center study performed by Ehehalt et al., only dealing with patients who acquired their infection within Europe, 10 patients with visceral leishmaniasis were identified with an age range between 1 and 79 years and the median age was 67 years. Three of the 10 identified patients with visceral leishmaniasis had a drug-induced immunosuppression (24). However, a study performed in a children’s hospital in Tehran has shown that the mean age of the 34 reported children suffering from visceral leishmaniasis was 26.9 months with an age range of 6–92 months, more than 90% were under the age of 5 years (1). Obviously, if visceral leishmaniasis occurs in childhood, it affects especially children younger than 5 years. The children reported in our case report were under the age of 5 years, and the child reported in the study of Calderaro et al. (25) was 2 years old.

In both patients, we decided to extend the therapy with liposomal amphotericin B up to 10 days. According to the current German guidelines, *Leishmania* acquired in the so-called “Old World” (Africa, Europe, Asia) should be treated with intravenous liposomal Amphotericin B at days 1–4 and at day 10 using 3 mg/kg/day, whereas *Leishmania* acquired in the so-called “New World” (North and South America) should be treated with intravenous 3–4 mg/kg/day for 10 days. As indicated by Di Martino et al., both therapies are effective in children (10). The regional differences in treatment intensity of *Leishmania* may be explained in part by different studies which have shown that *Leishmania* acquired in Brazil or in the Mediterranean basin may need higher total doses of liposomal amphotericin B than *Leishmania* acquired in India (31–36). However, it has been shown that short therapy courses with the same total dose of liposomal amphotericin B may be effective in Mediterranean visceral leishmaniasis as well (37). For economic reasons, in developing countries, oral miltefosine may be the treatment of choice because of lower costs, the possibility of oral treatment, and effectiveness (12). According to the current German guidelines, therapy with miltefosine is allowed in children ≥3 years of age. 1.5–2.5 mg/kg/day miltefosine should be taken for 28 days. Because of the severe and delayed course of disease in our two children, we decided to follow the guidelines for *Leishmania* acquired in the “New World.”

## Concluding Remarks

In conclusion, the reported cases shall illustrate the unspecificity of presentation of a well-known disease on the one hand and focus on specific, yet subtle bone marrow findings on the other hand. Therefore, in pancytopenia, a thorough analysis of the bone marrow beyond the common focus on malignancy also searching for parasites, such as *Leishmania*, is important. Due to mechanisms like polyclonal B cell activation, many serologic tests might be positive leading to false conclusions yet early initiation of a *Leishmania* serology when pancytopenia is present might help to accelerate establishing a diagnosis.

Leishmaniasis is to be included in the diagnostic considerations in patients presenting with persistent pancytopenia, and a positive travel history to endemic areas, as an effective therapy of this potentially life-threatening disease is available.

## Patient Consent

Family of patient A was informed and agreed upon publishing the data; family of patient B was lost of follow-up and therefore no consent could be obtained.

## Conflict of Interest Statement

The authors declare that the research was conducted in the absence of any commercial or financial relationships that could be construed as a potential conflict of interest.
